# Immunomodulatory Effects of Domoic Acid Differ Between *In vivo* and *In vitro* Exposure in Mice

**DOI:** 10.3390/md6040636

**Published:** 2008-12-22

**Authors:** Milton Levin, Heather Leibrecht, James Ryan, Frances Van Dolah, Sylvain De Guise

**Affiliations:** 1 Department of Pathobiology and Veterinary Science, University of Connecticut, Storrs, CT, 06269, USA; 2 Marine Biotoxins Program, NOAA/National Ocean Service, Center for Coastal Environmental Health and Biomolecular Research, 219 Fort Johnson Road, Charleston, SC 29412, USA

**Keywords:** Domoic acid, immunotoxicity, innate immunity, adaptive immunity

## Abstract

The immunotoxic potential of domoic acid (DA), a well-characterized neurotoxin, has not been fully investigated. Phagocytosis and lymphocyte proliferation were evaluated following *in vitro* and *in vivo* exposure to assay direct vs indirect effects. Mice were injected intraperitoneally with a single dose of DA (2.5 μg/g b.w.) and sampled after 12, 24, or 48 hr. In a separate experiment, leukocytes and splenocytes were exposed *in vitro* to 0, 1, 10, or 100 μM DA. *In vivo* exposure resulted in a significant increase in monocyte phagocytosis (12-hr), a significant decrease in neutrophil phagocytosis (24-hr), a significant decrease in monocyte phagocytosis (48-hr), and a significant reduction in T-cell mitogen-induced lymphocyte proliferation (24-hr). *In vitro* exposure significantly reduced neutrophil and monocyte phagocytosis at 1 μM. B- and T-cell mitogen-induced lymphocyte proliferation were both significantly increased at 1 and 10 μM, and significantly decreased at 100 μM. Differences between *in vitro* and *in vivo* results suggest that DA may exert its immunotoxic effects both directly and indirectly. Modulation of cytosolic calcium suggests that DA exerts its effects through ionotropic glutamate subtype surface receptors at least on monocytes. This study is the first to identify DA as an immunotoxic chemical in a mammalian species.

## 1. Introduction

Certain species of the marine diatom *Pseudo-nitzschia* produce the neurotoxin domoic acid (DA), which fish and shellfish can concentrate, putting consumers of these species such as marine mammals and humans at risk for adverse effects during and subsequent to harmful algal bloom (HAB) or ‘red tide’ events. Over the last several decades, the frequency and global distribution of HAB incidents appear to have increased and may be related to human activity, such as increased pollution runoff into aquatic ecosystems or global warming [1]. HAB toxins, including brevetoxin, microcystin, and saxitoxin, are immunotoxic in humans, mice, and aquatic mammals [2–5]. DA is immunotoxic in oysters and mussels [6, 7]. However, the potential immunotoxicity of DA in mammals has not been investigated. As human and wildlife exposure to DA is expected to continue, a better understanding of the sub-lethal effects of DA, as well as other HAB toxins, is warranted.

In humans, DA is the causative agent of amnesic shellfish poisoning (ASP) with symptoms including nausea, vomiting, diarrhea, dizziness, seizures and permanent loss of short term memory [8]. In 1987, over 100 people became ill and 4 people died after eating DA-contaminated mussels originating from Prince Edward Island [9]. Since 1998, hundreds of California sea lion deaths were linked to exposure to DA, resulting from the trophic transfer of DA from diatoms to prey such as northern anchovy during DA HAB events [10–12]. The rate of re-stranding following treatment for DA exposure was approximately 20 times higher than for animals stranded for other reasons, suggesting chronic changes may have affected their survival [12]. Sub-clinical DA-induced immunomodulation may have predisposed those animals to such chronic manifestations.

DA is a rigid analog of the neurotransmitter glutamate and a potent agonist of kainate and alpha amino-5-methyl-3-hydroxyisoxazolone-4-propionate (AMPA) subtypes of the glutamate receptor [13]. Persistent activation of these receptor subtypes results in rapid excitotoxicity, with the secondary activation of N-methyl-D-aspartate (NMDA) glutamate receptors and voltage dependent calcium channels, which leads to calcium dependent cell death and neuronal lesions in areas of the brain where glutamatergic pathways are heavily concentrated [14, 15].

Recent evidence emerged that glutamatergic pathways also exist in non-neuronal tissues [15–20]. For immune cells, NMDA receptors were identified in rat macrophages [21], while both NMDA and kainate/AMPA subtypes were detected in human and rodent lymphocytes [22–25] and were suggested to play a role in cell signaling and functional events, such as cell division.

The weight of evidence suggest that DA may be immunotoxic in mammals, as (1) DA was shown to be immunotoxic in bivalves [6, 7], (2) glutamate receptors (similar to receptors implicated in mediating neurotoxic effects) were demonstrated on mouse and human immune cells [22–25], and (3) exposure to glutamate modulated human mitogen-induced lymphocyte proliferation [24]. The hypothesis for the present study is that DA is immunotoxic in a mammalian species following both *in vitro* and *in vivo* exposure as measured by changes in immune cell functions. *In vitro* studies were performed to determine the direct effects, i.e. without the potential influence of administration, distribution, metabolism or excretion (ADME), of DA on immune cells and their functions by co-incubating isolated immune cells with DA. *In vivo* studies were performed to determine the potential indirect effects of DA on immune cells and their functions, i.e. with the potential influence of ADME and/or complex system interactions, for example, between the nervous and immune systems.

Assays to measure immunotoxicity included peripheral blood leukocyte phagocytosis and mitogen-stimulated lymphocyte (splenocyte) proliferation upon DA exposure. Both functional immune assays were validated by and are part of the National Toxicology Program to predict the immunotoxicity of chemicals [26, 27]. Phagocytosis is a key innate immune function performed predominately by peripheral blood neutrophils and monocytes, providing the first line of defense against invading organisms, particularly bacteria. Lymphocyte proliferation is a key adaptive immune response in which lymphocytes proliferate upon stimulation by an antigen or mitogen and represents the first step towards the production of effector and memory B and T lymphocytes.

DA has not been reported to bind directly to immune cells, however, as DA is an analog of glutamate, it is possible that it binds to immune cell glutamate receptor(s) in order to mediate immune functions. Modulation of cytosolic calcium is a mechanism involved in DA-induced neurotoxicity and involves cell membrane ionotropic glutamate receptors, such as AMPA and kainate receptors [15, 20]. In an initial attempt to identify the most likely cell surface glutamate receptor(s) by which DA modulates immune functions, qualitative changes in cytosolic calcium mobilization induced by several ionotropic glutamate receptor agonists were compared to that of DA. This study provides the foundation to determine the immunotoxicity of DA (the hazard identification step in risk assessment) and elucidate the mechanisms and pathways involved in the response to toxic levels of DA in mammals, which may be relevant to marine mammals and humans with documented DA exposure.

## 2. Results

### 2.1 Leukocyte flow cytometry profile

**Figure 1.**
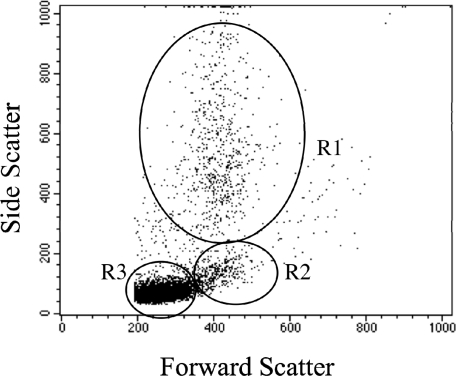
Flow cytometric dot plot of mouse peripheral blood leukocytes. Sub-populations of leukocytes can be easily distinguished based on forward scatter (relative size) and side scatter (relative complexity). Neutrophils (R1) are large and complex; lymphocytes (R3) are smaller and less complex, while monocytes (R2) are slightly larger than lymphocytes and less complex than neutrophils.

A representative scatterplot of the flow cytometric profile of mouse peripheral blood leukocytes is shown in Figure 1. The different sub-populations of cells were easily distinguished on the basis of relative cell size (forward scatter) and complexity (side scatter). Neutrophils were large and complex (granular), lymphocytes were small and less complex, and monocytes were slightly larger than lymphocytes and less complex than neutrophils.

### 2.2 In vivo clinical signs

The *in vivo* study used a single i.p. injection of dose DA, 2.5 μg/g, previously shown in mice to be symptomatic but sub-lethal. In this study, two of the 30 mice exposed to DA showed minimal loss of balance for approximately 15 sec. Within 30 min after dosing, their behavior returned to normal. No other clinical signs were detected within the first hour following exposure and all mice appeared normal prior to euthanasia at all time points.

### 2.3 In vivo phagocytosis

**Figure 2.**
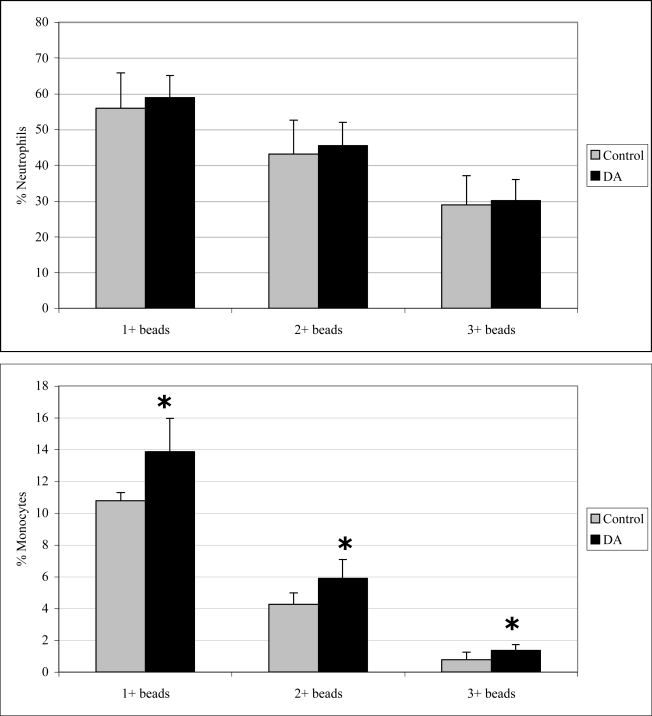
*In vivo* peripheral blood neutrophil (top) and monocyte (bottom) phagocytosis (mean +SD) in unexposed (control; n=10) and DA-exposed mice (n=10) 12 hr after exposure (t-test: *, p < 0.05).

**Figure 3.**
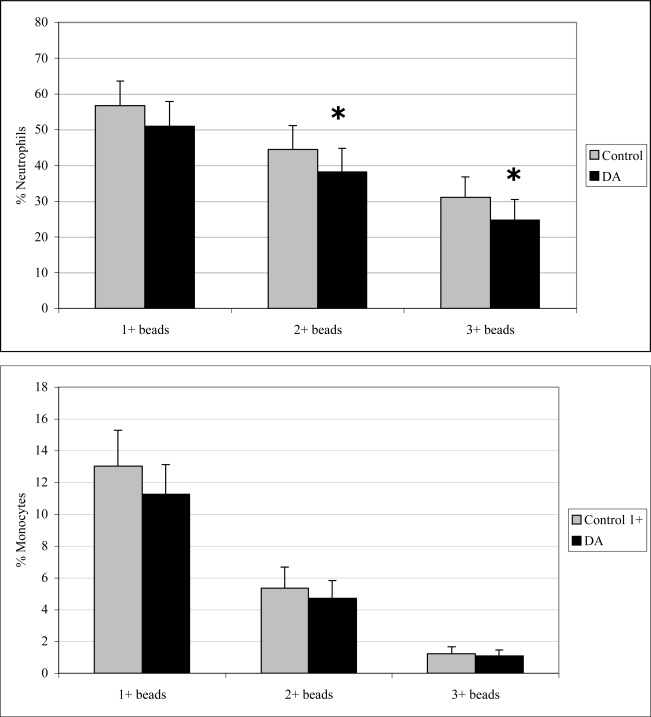
*In vivo* peripheral blood neutrophil (top) and monocyte (bottom) phagocytosis (mean +SD) in unexposed (control; n=10) and DA-exposed mice (n=10) 24 hr after exposure (t-test: *, p < 0.05).

**Figure 4.**
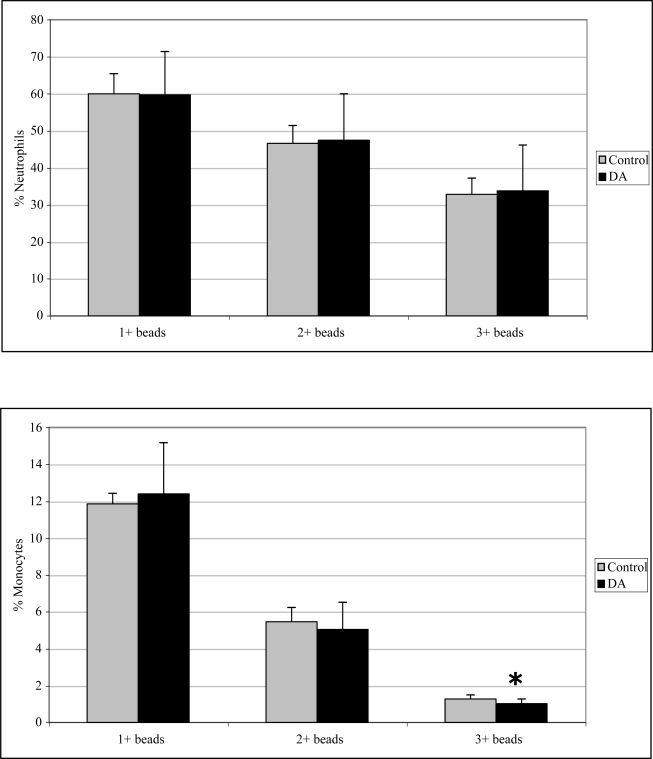
*In vivo* peripheral blood neutrophil (top) and monocyte (bottom) phagocytosis (mean +SD) in unexposed (control; n=10) and DA-exposed mice (n=10) 48 hr after exposure (t-test: *, p < 0.05).

Monocyte phagocytosis measured 12 hr after exposure was significantly increased in DA-exposed mice compared to control mice by 29, 38, and 77% for 1+, 2+, and 3+ beads, respectively (Figure 2). There were no effects on neutrophil phagocytosis at that time point. Neutrophil phagocytosis measured 24 hr after exposure was significantly decreased in DA-exposed mice compared to control mice by 14 and 21% for 2+ and 3+ beads, respectively (Figure 3). There were no effects on monocyte phagocytosis at that time point. Monocyte phagocytosis measured 48 hr after exposure was significantly decreased in DA-exposed mice compared to control mice by 20% for 3+ beads (Figure 4). There were no effects on neutrophil phagocytosis at that time point.

### 2.4 In vivo lymphocyte proliferation

**Figure 5.**
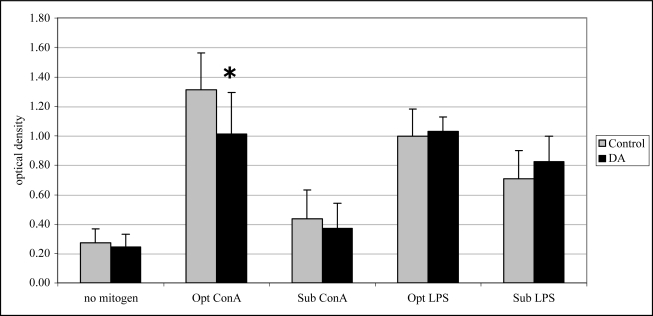
*In vivo* mitogen-induced lymphocyte (splenocyte) proliferation (mean +SD) in unexposed (control; n=10) and DA-exposed mice (n=10) 24 hr after exposure (t-test, *p < 0.05).

Twenty-four hr after exposure, T cell proliferation (with optimal ConA) was significantly reduced (33%) compared to control mice (Figure 5). Mitogen-induced lymphocyte proliferation was not significantly affected after 12 and 48 hr exposure to DA (data not shown).

### 2.5 In vitro phagocytosis

**Figure 6.**
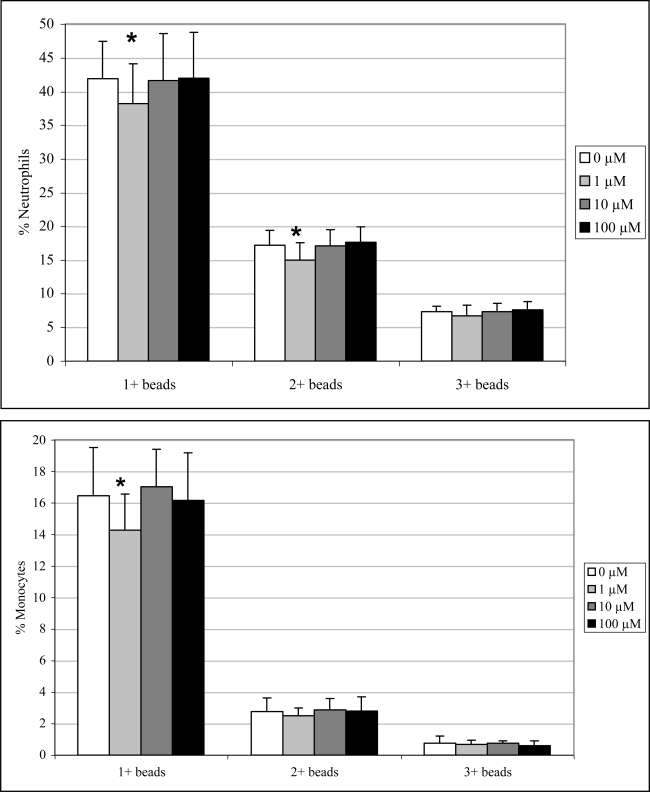
*In vitro* peripheral blood neutrophil (top) and monocyte (bottom) phagocytosis (mean +SD) with increasing concentrations of DA (n=10; RM ANOVA, *p < 0.05).

Exposure to 1μM DA significantly decreased neutrophil and monocyte phagocytosis (1+ beads only) by 9 and 13%, respectively, and neutrophils phagocytosis (2+ beads only) by 13 % (Figure 6). No effects were observed at the higher concentrations.

### 2.6 In vitro lymphocyte proliferation

**Figure 7.**
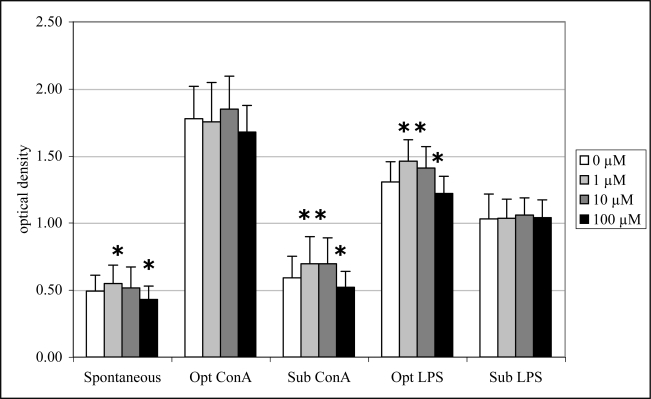
*In vitro* mitogen-induced lymphocyte (splenocyte) proliferation (mean +SD) with increasing concentrations of DA (n=10; RM ANOVA, *p < 0.05).

Exposure to 1 μM DA significantly increased spontaneous (12%), sub-optimal ConA-induced (18%) and optimal LPS-induced lymphocyte proliferation (12%) while exposure to 10 μM DA significantly increased only sub-optimal ConA-induced (18%) and optimal LPS-induced (8%) lymphocyte proliferation (Figure 7). Exposure to 100 μM DA, in contrast, significantly decreased spontaneous (12%), sub-optimal ConA-induced (12%) and optimal LPS-induced (7%) lymphocyte proliferation (Figure 7).

### 2.7 Mobilization of calcium in blood leukocytes

**Figure 8.**
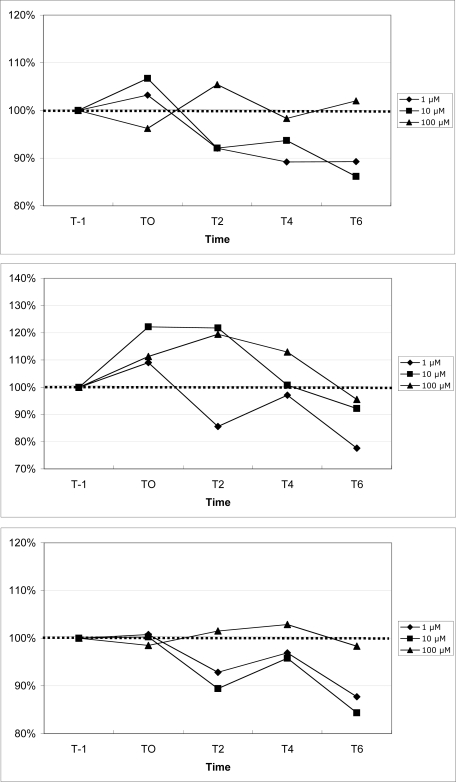
Changes in peripheral blood neutrophil (top), monocyte (middle), and lymphocyte (bottom) cytosolic calcium upon exposure to increasing concentrations of domoic acid. Data are expressed as the % of the unexposed control over time (T-1: 1 min prior to exposure, T0: time of exposure to agonists, T2: 2 min after exposure, T4: 4 min after exposure, T6: 6 min after exposure). For each time point, data are presented as the average of 3 mice. (100% indicated by dotted line)

Mouse mean peripheral blood neutrophil, monocyte and lymphocyte fluorescence increased by 407%, 408%, and 408%, respectively, 1 min following exposure to ionomycin (data not shown), suggesting appropriate cell loading with the probe. The results for calcium mobilization upon exposure to increasing concentrations of DA are shown in Figure 8. For all three sub-populations of leukocytes, 1 μM DA consistently reduced cytosolic calcium, as measured by a reduction of cell fluorescence compared to baseline fluorescence. For neutrophils and lymphocytes, 10 μM DA also reduced cytosolic calcium, while calcium was moderately increased in monocytes. 100 μM DA moderately increased cytosolic calcium in monocytes, while calcium was modestly increased in neutrophils and lymphocytes.

**Figure 9.**
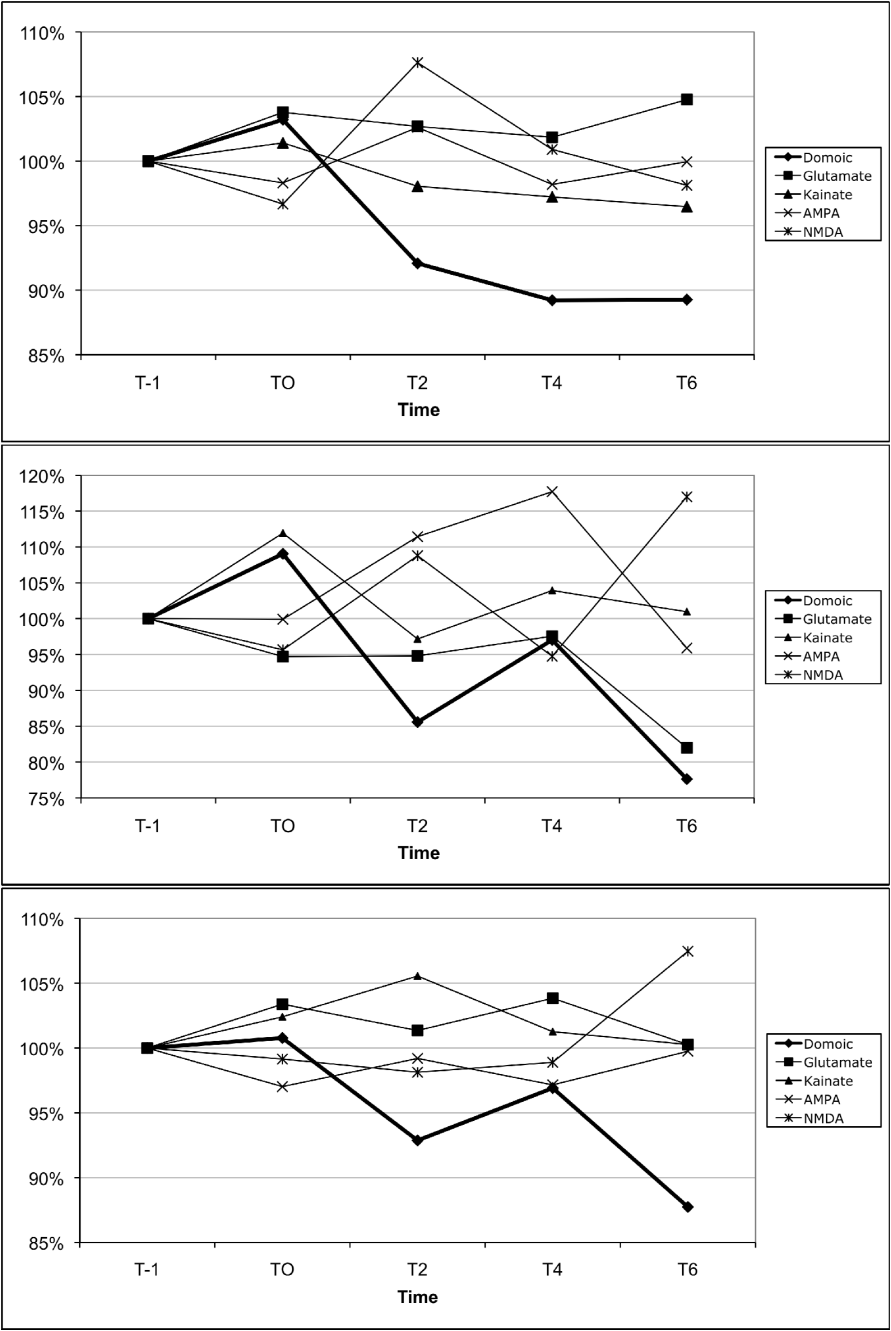
Changes in peripheral blood neutrophil (top), monocyte (middle), and lymphocyte (bottom) cytosolic calcium upon exposure to the ionotropic glutamate receptor agonists, L-glutamate, kainate, AMPA, NMDA, as well as domoic acid (all at 1 μM). Data are expressed as the % of the unexposed control over time (T-1: 1 min prior to exposure, T0: time of exposure to agonists, T2: 2 min after exposure, T4: 4 min after exposure, T6: 6 min after exposure). For each time point, data are presented as the average of 3 mice per agonists. (100% indicated by dotted line)

The results for calcium mobilization upon exposure to the different glutamate receptor agonists in comparison to DA are shown in Figure 9. For all three sub-populations of leukocytes, DA consistently reduced cytosolic calcium, as measured by a reduction in cell fluorescence compared to baseline fluorescence. The only agonists that also induced a consistent reduction in cytosolic calcium in neutrophils, monocytes, and lymphocytes (Figure 8) were kainate, L-glutamate, and AMPA, respectively. However, the magnitude of the change induced by those agonists was consistently less than that induced by DA for neutrophils and lymphocytes.

## 3. Discussion and Conclusion

To the authors’ knowledge, this is the first report to demonstrate the immunotoxic effects of DA in any mammalian species. Previous reports demonstrated that DA was not immunotoxic either upon i*n vitro* exposure of isolated human dendritic cells [28] or rats exposed *in vivo* [29], although different cell types and route of exposure were employed. When exposed to physiologically relevant concentrations of DA, different responses were observed between *in vivo* and *in vitro* exposure. This may reflect differences in exposure times, ADME (especially excretion, see below) and the potential for both direct and indirect effects of DA on immune cells following *in vitro* and *in vivo* exposure.

### 3.1 In vivo exposure

*In vivo* exposure to DA modulated both innate and adaptive immune cell functions and effects appeared to be cell type- and time-specific. In general, it appeared that *in vivo* DA exposure acutely enhanced innate immune function (monocyte phagocytosis) by 12 hr, followed by immune suppression of both innate (neutrophil phagocytosis) and adaptive (T lymphocyte proliferation) immune functions by 24 hr, and with return to control levels 48 hr after exposure. This time course correlates well with the clearance of DA from mouse blood, with almost 99% clearance by 24 hr [30, 31].

In a similar study following *in vivo* DA exposure, gene expression in mouse peripheral blood leukocytes was assayed (Ryan, J., personal communication). Several chemokines were found to be differentially expressed, including the down regulation of Ccl5/Rantes, which was also shown to be down regulated in monocytes exposed to glucocorticoids *in vitro* [32]. In addition, DA exposure *in vivo* also produced an up regulation in integrin alpha 4, which was shown to be important in recruitment of mononuclear cells to sites of inflammation but also necessary for immune cells to permeate the CNS [33]. Interestingly, isolated rat astrocytes exposed to 10 μM DA showed up regulation of chemokine genes for IL-1α, IL-1β, IL-6 [34]. The relationships between changes in immune cell gene expression and immune functions, which may be related to or independent of glucocorticoid and glutamate receptors, warrant future studies.

### 3.2 In vitro exposure

*In vitro* exposure to DA directly modulated innate and adaptive immune cell functions, although the effects were different (direction of change and affected cell types) from *in vivo* exposure. Neutrophil (1 and 2 or more beads) and monocyte (1 or more beads) phagocytosis were significantly reduced only at the lowest concentration tested. It is possible that the most active phagocytic cells (3+) may not have been the preferential targets of DA. Lymphocyte proliferation, including spontaneous as well as T and B cell induced, was increased at low concentrations and decreased at high concentrations (further discussed below). Hormetic immune responses are not unusual following exposure to various endogenous and exogenous chemicals [35, 36]. T cell proliferation was significantly modulated by DA using only the sub-optimal concentration of ConA. In this case, lymphocytes that were proliferating at a slower rate proved to be more sensitive to the immunotoxic effects of DA than lymphocytes proliferation at a higher rate. Importantly, this effect would have been missed if only using the optimal ConA mitogen concentration. This highlights the need to test both suboptimal and optimal concentrations of mitogens when assessing the immunotoxicity of chemicals on lymphocyte proliferation [37].

### 3.3 Potential mechanism(s)/pathways(s) mediating in vitro DA-induced immunotoxicty

Experiments were initiated to determine the mechanisms and pathways by which DA may exert its immunotoxic effects. Experiments were performed with limited numbers of mice (n=3) to assess qualitative trends in modulation of cytosolic calcium by DA, a well described effect documented in various neural and non-neural cells [15], to determine whether the presence of glutamate receptors (and their different sub-types) on immune cells may be involved in DA-induced immunotoxicity.

DA exposure resulted in qualitative reductions and increases in cytosolic calcium compared to unexposed control in all three leukocyte subtypes tested in the present study. Numerous reports have documented increases in cytosolic calcium in different cell types (see review by Pulido, 2008). Similarly, cytosolic calcium slightly increased in all cell types upon exposure to 100 μM DA, with the greatest increase in monocytes. However, upon exposure to 1 μM (for all cell types) and 10 μM (for neutrophils and lymphocytes), cytosolic calcium was decreased. In this case, DA may have induced calcium sequestration into the endoplasmic recticulum and/or mitochondria, a response documented by glutamate receptor agonist [38–40].

For all three immune cell subtypes, changes in cytosolic calcium were compared to changes in immune functions (upon *in vitro* exposure) in an initial attempt to explain DA-induced immunotoxicity. For neutrophils and monocytes, phagocytosis was significantly reduced only at 1 μM DA, the same concentration that also reduced cytosolic calcium in both cell types. As calcium mobilization is necessary for phagocytosis [41, 42], the lack of free cytosolic calcium may help explain the reduction in phagocytosis. Although calcium was reduced by 10 μM in both cell types, no significant changes in phagocytosis were observed, an observation not easily explained at this time. At 100 μM, no significant changes in phagocytosis were observed and corresponded with modest to moderate increases in calcium in neutrophils and monocytes, respectively, suggesting that these increases in calcium were not involved in modulating phagocytosis.

Limited data exists for glutamate receptors on differentiated monocytes. U937 cells (human histiocytic lymphoma derived) demonstrated differential growth and morphology upon exposure to glutamate receptor agonists depending on external glutamate concentrations [25]. In primary rat microglial cells [43], kainate induced rapid redistribution of the actin cytoskeleton, a necessary step in the process of phagocytosis. NMDA receptors have been identified on rat macrophages [21]. Our results also demonstrated a consistent reduction in cytosolic calcium upon stimulation with L-glutamate receptors in monocytes that was consistent with that for DA (at least 4 and 6 min. post-exposure), suggesting the presence and functionality of Glutamate-responsive receptors on mouse monocytes, and the possibility that they could be involved in mediating the toxicity of DA on those cells. Taken together, these data suggest that glutamate receptors exist on monocytes and may be involved in modulating phagocytosis. Nevertheless, the presence and potential role of glutamate receptors responsive to DA on neutrophils are unknown at this time, and none of the receptor subtypes matched the direction and magnitude of change in cytosolic calcium obtained with DA.

For both T and B lymphocyte, 100 μM DA significantly reduced lymphocyte proliferation, which corresponded with a very modest increase in calcium mobilization. Although increases in cytosolic calcium have been show to reduce lymphocyte proliferation [44], it is not possible to conclude that this was the case in this study. Interestingly, 1 and 10 μM DA significantly increased proliferation, which corresponded with reduced cytosolic calcium in peripheral blood lymphocytes. The role of reduced calcium in mediating proliferation appears unlikely. Alternatively, modulation of voltage-activated potassium channels by glutamate [45] may be one mechanism that explains both enhancement and suppression of T lymphocyte proliferation. Poulopoulou et al. (2005) demonstrated that low glutamate concentrations (below 100 μM) positively modulated potassium channel gating resulting in T lymphocytes that were readily responsive to stimuli with a maximal effect at 1 μM. In contrast, glutamate at concentrations >100 μM was shown to decrease potassium channel currents thereby inhibiting T lymphocyte responsiveness to stimuli. The previous study may help explain how the low and medium concentrations of DA enhanced lymphocyte proliferation while high concentrations of DA reduced proliferation in the current study. Although no report has demonstrated the effects of glutamate on B cells, they may share common mechanisms and pathways with T cells. Taken together, these data support that glutamate receptors may be involved in modulation of lymphocyte proliferation, but our studies have not identified a receptor sub-type that could match the magnitude of cytosolic calcium mobilization induced by DA.

Though studies have identified glutamate receptor subtypes on human and rodent immune cells [22–24], clearly, additional work must be performed to confirm that DA can exert its immunomodulatory effects through these receptors.

### 3.4 Differences between direct vs indirect effects

There are two possible explanations to account for differences in DA-induced immunotoxicity between *in vitro* and *in vivo* exposure. First, *in vitro* exposure was carried out at fixed concentrations for the entire incubation period, whereas following *in vivo* exposure, immune cells would not be exposed to prolonged high concentrations due to the rapid renal excretion of DA, which reduces the ultimate concentration of DA in blood to which immune cells are exposed. Therefore, the final concentration of DA ‘seen’ by immune cells would differ between *in vitro* and *in vivo* exposure.

Second, modulation of immune functions *in vivo* may be secondary to the direct effects of DA on other organ systems. There is clear evidence that the central nervous system modulates the peripheral immune system through the hypothalamic-pituitary-adrenal (HPA) axis [46]. It is possible that DA binding to glutamate receptors in the hippocampus, the well-described mechanism by which DA exerts its neurotoxic effects [47, 48], may activate the HPA axis, resulting in release of adrenocorticotropic hormone (ACTH). Microinjection of agonists to hippocampal glutamate receptors, including the AMPA subtype receptor which DA can bind, induced elevation of plasma ATCH in a dose-dependent fashion [49]. ACTH, in turn, induces adrenal release of glucocorticoids, which modulate innate and adaptive immune responses [50–52]. Glucocorticoids exert their effects by binding to the cytosolic glucocorticoid receptor (GC), a ligand-dependent transcription factor, which regulates gene expression and functions in immune cells. For example, human monocytes exposed *in vitro* to glucocorticoids resulted in an induction of “phagocytic” genes and was associated with an approximate 2.5-fold increase in phagocytosis of fluorescent latex beads and complement opsonized *Leishmania major*, compared to controls [32]. In the present study, monocyte phagocytosis of fluorescent latex beads was enhanced at 12 hr. Glucocorticoids were shown to suppress neutrophil phagocytosis [50, 53] and suppress lymphocyte proliferation [54, 55], similar to the effects observed in the present study. Gene expression changes seen in the brain after DA exposure are consistent with an increase in glucocorticoid production with the up-regulation of serum and glucocorticoid kinase, and Gilz (glucocorticoid induced leucine zipper) [56]. Further in the same study, several immune relevant genes were differentially expressed in the brain such as cyclooxygenase 2, CSA-conditional T cell activation dependent protein, and alpha and beta subunits of cytotoxic T lymphocyte-associated protein 2. In the current study, glucocorticoid levels were not measured, but should be explored in future experiments to help elucidate indirect mechanisms upon *in vivo* DA exposure.

Different glutamate receptor types/expression/activity between neutrophils, monocytes, and T lymphocytes could help explain why monocytes were acutely sensitive to the effects of DA, while the response by neutrophils and T lymphocyte was delayed. Interestingly, no effects were observed for B lymphocytes, suggesting differences in surface receptors and/or signaling pathways among immune cells.

### 3.5. Relevancy of immune modulation

Any modulation of immune functions, whether in increase or decrease, is of concern. Although the magnitudes of the changes in immune functions were sometimes small, these changes could have significant biological consequences [57]. Reduction of phagocytosis may lead to decreased pathogen clearance, allowing opportunistic pathogens to produce disease [58, 59]. Enhancement of phagocytosis could lead to premature release of cytosolic lysosomal content and reactive oxygen species, resulting in local inflammation and tissue damage [60]. Non-specific and unregulated increases in lymphocyte proliferation may be the initiating step in the transformation of a lymphocyte into a cancer cell [61]. Interestingly, glutamate receptors antagonists have been shown to be important in the suppression of some tumors [62, 63], while DA has been show to increase chromosomal abnormalities in the Caco-2 cell line [64], suggesting a potential role of DA in tumor formation. In addition, non-specific stimulation of lymphocyte proliferation may potentially contribute to autoimmune diseases or anergy, an active state of unresponsiveness [61]. A reduction in lymphocyte proliferation may prevent the expansion of effector and memory T and B lymphocytes, thus increasing an individual’s susceptibility to opportunistic infection or neoplasms [65].

### 3.6 Conclusion

This is the first study to demonstrate the immunomodulatory effects of both *in vivo* and *in vitro* exposure to DA in a mammalian species. In the risk assessment scheme, DA can be viewed as a hazard, requiring additional studies, including elucidating the mechanism(s) of action. The present study provides initial data to suggest that calcium mobilization and glutamate sub type cell surface receptors as potential mechanisms and pathways involved in the response to toxic levels of domoic acid in mammals. In addition, future gene expression profiles may help identify specific biomarkers in blood that may lead to a biomonitoring system for subacute exposure in humans and protected marine species whose populations are exposed annually to toxic algal blooms.

## 4. Experimental Section

### 4.1 In vivo exposure

Domoic acid (DA; Sigma, St Louis, MO) was re-suspended in sterile phosphate buffered saline (PBS) at 0.5 μg/μL. Adult, 25–28 g ICR female mice (Harlan, Indianapolis, IN) were weighed and received an intraperitoneal (i.p.) injection of 2.5 μg/g DA (n=10 mice per exposure period) or vehicle control (PBS; n=10 mice per exposure period) with a U-100 Insulin syringe and a 28 gauge needle. This dose was chosen as it was previously shown in mice to be symptomatic but sub-lethal (Ryan, personal communication) [30, 56], as well as inducing changes in brain gene expression [30]. Mice were observed for one hr after dosing for any clinical signs of acute DA toxicity. At the end of each exposure period (12, 24 or 48 hr), mice were euthanized by CO_2_ inhalation. Blood was immediately collected via cardiac puncture followed by cervical dislocation to ensure death, and spleen was removed and stored in ice cold DMEM until processing (below). The study design and procedures were approved by the Institutional Animal Care and Use Committee (IACUC) at the University of Connecticut.

### 4.2 Phagocytosis

From individual mouse whole blood samples, erythrocytes were lysed using NH_4_Cl and the leukocytes were re-suspended in Hanks Balanced Salt Solution (HBSS, Gibco BRL, Grand Island, NY). Cells were washed twice with HBSS, and their viability was assessed using the exclusion dye trypan blue. Leukocytes were adjusted to 2 × 10^6^/ml in HBSS and plated (100 μl per well) in a round bottom 96-well plate (Falcon, Becton Dickinson, Lincoln Park, NJ) in triplicate. One μm-diameter fluorescent latex beads (Molecular Probes, Eugene, OR) were added to the cell suspension to obtain a ratio of approximately 100 beads/cell, and cells were incubated for one hr at 37°C, under agitation at 300 rpm using a Thermomixer R (Eppendorf, Hamburg, Germany). The cell suspension from each well was then layered on a cushion of ice cold 3% bovine serum albumin (Sigma, St. Louis, MO) and centrifuged at 150g for 8 min at 4°C. The supernatant containing the free beads was discarded and the cells were re-suspended in 200 μl of PBS containing 1% neutral buffered formalin (Decal Corp, Tallman, NY). Cells were stored at 4°C until analysis (within 24 hr). The fluorescence of approximately 10,000 cells was read with a FACScan (Becton Dickinson, Rutherford, NJ) flow cytometer using the CellQuest software (Becton Dickinson Immunocytometry System, San Jose, CA). Neutrophils and monocytes were gated electronically according to their relative size (forward scatter; FSC) and complexity (side scatter; SCC). The fluorescence of the cells was read at 530 nm (FL-1) on a logarithmic scale using the fluorescence of free beads as reference. Cells acquired a fluorescence equal to that of the number of beads they ingested. Phagocytosis was evaluated as the proportion of neutrophil and monocytes that had phagocytized one or more beads (1+, the proportion of all cells that participate in phagocytosis, includes 2+ and 3+), two or more beads (2+, includes 3+) and three or more beads (3+, the proportion of cells that are most efficient in phagocytosis), the endpoint of phagocytosis routinely reported [66–68].

### 4.3. Lymphocyte proliferation

From each individual spleen, a single cell suspension was prepared using two pairs of forceps in complete Dulbecco’s modified eagle medium (DMEM, Gibco BRL, Grand Island, NY) supplemented with (all from Gibco BRL, Grand Island, NY) 1 mM sodium pyruvate, 100 μM non-essential amino acids, 25 mM HEPES, 2 mM L-glutamine, 100 U/ml penicillin and 100 μg/ml streptomycin, along with 10 % fetal bovine serum (Hyclone, Logan, UT), hereafter referred to as complete DMEM. Mononuclear cells were isolated by density gradient centrifugation on Ficoll-Paque plus (Amersham Biosciences, Uppsala Sweden) for 35 min at 990 g. The mononuclear cells were re-suspended in complete DMEM, washed once, and enumerated with their viability assessed using the exclusion dye trypan blue. Lymphocytes in complete DMEM were plated (1 × 10^6^ cells/ml final concentration, 100 μl per well) in triplicate in 96 well flat bottom tissue culture plates (Falcon, Becton Dickinson, Franklin Lakes, NJ). Cells were incubated at 37°C with 5% CO_2_ for a total of 66 hr with the T cell mitogen concanavalin A (Con A Sigma, St. Louis, MO) or the B cell mitogen lipopolysaccharide (LPS, Sigma, St. Louis, MO, USA). Con A was used at a sub-optimal concentration (0.1 μg/ml), as well as at an optimal concentration (1 μg/ml). LPS was used at a sub-optimal concentration (0.05 μg/ml), as well as at an optimal concentration (5 μg/ml). Suboptimal concentrations were used as they proved more sensitive in detecting immunotoxicity [37]. Lymphocyte proliferation was evaluated as the incorporation of 5-bromo-2’-deoxyuridine (BrdU), a thymidine analogue, added for the last 18 hr of incubation, and subsequently detected with a monoclonal antibody and a colorimetric enzymatic reaction (Cell Proliferation ELISA BrdU (colorimetric), Roche Diagnostics GmbH, Mannheim Germany) as per manufacturer’s instructions using an ELISA plate reader (Multiskan EX v.1.0) at 690 nm with a reference wavelength of 450 nm.

### 4.4 In vitro exposure

In a separate experiment, individual blood samples (collected via cardiac puncture) and spleens from 10 adult ICR female mice were collected immediately after euthanasia, and processed as above, then incubated with domoic acid *in vitro*. Leukocytes or lymphocytes from the same individual were exposed to DA *in vitro* at 4 concentrations: 1, 10, and 100 μM DA or vehicle only (0 μM). The 1 μM dose was chosen to simulate the blood concentration of DA found in mice two hr after i.p. injection of 2 μg/g [31]. The 10 μM dose and 100 μM dose approximate concentrations of DA that blood cells may experience at early time points (30 and 10 min, respectively) following i.v. injection of 2 μg/g of DA [69], a dose slightly below the *in vivo* dose employed in the current work. Assays for phagocytosis and lymphocyte proliferation were performed as described above.

### 4.5 Cytosolic calcium mobilization

In separate experiments, peripheral blood leukocytes were collected, adjusted to 2 × 10^6^/ml, and incubated with the fluorescent Ca^2+^ probe, Fluo-3/acetoxymethyl (3 mM, Molecular Probes, Eugene, OR) in a 0.1% bovine serum albumin (Sigma, St. Louis, MO)/HBSS solution at 37°C for 30 min. Cells were centrifuged for 10 min at 150 g, re-suspended in HBSS and further incubated at 37°C for an additional 30 min (to ensure cleavage of the acetoxymethyl from the probe by non-specific esterases). Cells were centrifuged for 10 min at 150 g and re-suspended in 1.6 mM CaCl_2_ (Sigma, St. Louis, MO)-HBSS at room temperature.

To ensure proper loading of cells with the probe, the fluorescence of a sub-sample of leukocytes was read with a FACScan (Becton Dickinson, Rutherford, NJ) flow cytometer using the CellQuest software (Becton Dickinson, Immunocytometry System, San Jose, CA) at 530 nm (FL-1) for 30 sec, followed by the addition of 1μM ionomycin (Molecular Probes, Eugene, OR), an ionophore used to increase cytosolic calcium concentrations. The fluorescence was recorded for an additional 3 min.

The basal fluorescence of leukocytes was read with a FACScan flow cytometer at 530 nm (FL-1) for 10 sec. Cells were exposed to 0, 1, 10, and 100 μM DA. Cell fluorescence was recorded for 10 sec 1 min before (baseline fluorescence) and every 2 min (for 6 min) immediately following the addition of DA (time 0). Glutamate receptor agonists, kainate, AMPA, NMDA, and L-glutamate (all from Sigma, St. Louis, MO), were tested at 0 and 1μM, the same molar concentration shown to modulate phagocytosis upon *in vitro* exposure (see Results). It was reasoned that if the pattern of calcium mobilization for one or more of the selected agonists was similar to that produced by DA, it could be inferred that DA exerts its effects through that particular agonist receptor(s). Cell fluorescence was simultaneously recorded for all leukocytes. Neutrophils, monocytes, and lymphocytes were then analyzed separately based on electron gates. Data were expressed as the relative fluorescence (% unexposed) of exposed cells in time relative to the baseline fluorescence (T-1). Data were collected from three mice per agonists.

### 4.6 Statistics

For the *in vivo* experiment, within each time point (12, 24 or 48 hr), DA-exposed mice were compared to unexposed (control) mice using a Student’s t-test and p<0.05 for statistical significance. For the *in vitro* experiment, a repeated measures one-way analysis of variance (RM ANOVA) was performed to compare the exposed groups to the unexposed group using p<0.05 for statistical significance. All analyses were performed using the SigmaStat 3.5 (Systat, San Jose, CA) software. Cytosolic calcium mobilization experiments were performed with limited numbers of mice (n=3) to assess qualitative trends in modulation of cytosolic calcium by DA, therefore, no statistical analyses were performed.

## References

[1] Van Dolah FM (2000). Marine algal toxins: origins, health effects, and their increased occurrence. Environ. Health Perspect..

[2] Sayer A, Hu Q, Bourdelais AJ, Baden DG, Gibson JE (2005). The effect of brevenal on brevetoxin-induced DNA damage in human lymphocytes. Arch. Toxicol.

[3] Shen PP, Zhao SW, Zheng WJ, Hua ZC, Shi Q, Liu ZT (2003). Effects of cyanobacteria bloom extract on some parameters of immune function in mice. Toxicol. Lett.

[4] Walsh CJ, Luer CA, Noyes DR (2005). . Effects of environmental stressors on lymphocyte proliferation in Florida manatees, Trichechus manatus latirostris. Vet. Immunol. Immunopathol.

[5] Witkowski JM, Siebert J, Lukaszuk K, Trawicka L (1993). Comparison of effect of a panel of membrane channel blockers on the proliferative, cytotoxic and cytoadherence abilities of human peripheral blood lymphocytes. Immunopharmacol.

[6] Dizer H, Fischer B, Harabawy AS, Hennion MC, Hansen PD (2001). Toxicity of domoic acid in the marine mussel Mytilus edulis. Aquat. Toxicol.

[7] Jones TO, Whyte JN, Ginther NG, Townsend LD, Iwama GK (1995). Haemocyte changes in the Pacific oyster, Crassostrea gigas, caused by exposure to domoic acid in the diatom Pseudonitzschia pungens f. multiseries. Toxicon.

[8] Jeffery B, Barlow T, Moizer K, Paul S, Boyle C (2004). Amnesic shellfish poison. Food Chem. Toxicol.

[9] Perl TM, Bedard L, Kosatsky T, Hockin JC, Todd EC, Remis RS (1990). An outbreak of toxic encephalopathy caused by eating mussels contaminated with domoic acid. N. Engl. J. Med.

[10] Lefebvre KA, Powell CL, Busman M, Doucette GJ, Moeller PD, Silver JB, Miller PE, Hughes MP, Singaram S, Silver MW, Tjeerdema RS (1999). Detection of domoic acid in northern anchovies and California sea lions associated with an unusual mortality event. *Nat.*. Toxins.

[11] Scholin CA, Gulland F, Doucette GJ, Benson S, Busman M, Chavez FP, Cordaro J, DeLong R, De Vogelaere A, Harvey J, Haulena M, Lefebvre K, Lipscomb T, Loscutoff S, Lowenstine LJ, Marin R., Miller PE, McLellan WA, Moeller PD, Powell CL, Rowles T, Silvagni P, Silver M, Spraker T, Trainer V, Van Dolah FM (2000). Mortality of sea lions along the central California coast linked to a toxic diatom bloom. Nature.

[12] Gulland FM, Haulena M, Fauquier D, Langlois G, Lander ME, Zabka T, Duerr R (2002). Domoic acid toxicity in Californian sea lions (*Zalophus californianus*): clinical signs, treatment and survival. Vet. Rec.

[13] Hampson DR, Manalo JL (1998). The activation of glutamate receptors by kainic acid and domoic acid. Nat. Toxins.

[14] Berman FW, LePage KT, Murray TF (2002). Domoic acid neurotoxicity in cultured cerebellar granule neurons is controlled preferentially by the NMDA receptor Ca^2+^ influx pathway. Brain Res.

[15] Pulido OM (2008). Domoic acid toxicologic pathology: a review. Mar Drugs.

[16] Boldyrev AA, Carpenter DO, Johnson P (2005). Emerging evidence for a similar role of glutamate receptors in the nervous and immune systems. J. Neurochem.

[17] Skerry TM, Genever PG (2001). Glutamate signalling in non-neuronal tissues. Trends Pharmacol. Sci.

[18] Gill S, Veinot J, Kavanagh M, Pulido O (2007). Human heart glutamate receptors - implications for toxicology, food safety, and drug discovery. Toxicol. Pathol.

[19] Gill SS, Mueller RW, McGuire PF, Pulido OM (2000). Potential target sites in peripheral tissues for excitatory neurotransmission and excitotoxicity. Toxicol. Pathol.

[20] Gill SS, Pulido OM (2001). Glutamate receptors in peripheral tissues: current knowledge, future research, and implications for toxicology. Toxicol. Pathol.

[21] Dickman KG, Youssef JG, Mathew SM, Said SI (2004). Ionotropic glutamate receptors in lungs and airways: molecular basis for glutamate toxicity. Am. J. Respir. Cell Mol. Biol.

[22] Boldyrev AA, Kazey VI, Leinsoo TA, Mashkina AP, Tyulina OV, Johnson P, Tuneva JO, Chittur S, Carpenter DO (2004). Rodent lymphocytes express functionally active glutamate receptors. Biochem. Biophys. Res. Commun.

[23] Ganor Y, Besser M, Ben-Zakay N, Unger T, Levite M (2003). Human T cells express a functional ionotropic glutamate receptor GluR3, and glutamate by itself triggers integrin-mediated adhesion to laminin and fibronectin and chemotactic migration. J. Immunol.

[24] Lombardi G, Dianzani C, Miglio G, Canonico PL, Fantozzi R (2001). Characterization of ionotropic glutamate receptors in human lymphocytes. Br. J. Pharmacol.

[25] Haas HS, Pfragner R, Siegl V, Ingolic E, Heintz E, Schauenstein K (2005). Glutamate receptor-mediated effects on growth and morphology of human histiocytic lymphoma cells. Int. J. Oncol.

[26] Luster MI, Portier C, Pait DG, Rosenthal GJ, Germolec DR, Corsini E, Blaylock BL, Pollock P, Kouchi Y, Craig W, White KL, Munson AE, Comment CE (1993). Risk assessment in immunotoxicology. II. Relationships between immune and host resistance tests. Fundam. Appl. Toxicol.

[27] Luster MI, Portier C, Pait DG, White KL, Gennings C, Munson AE, Rosenthal GJ (1992). Risk assessment in immunotoxicology. I. Sensitivity and predictability of immune tests. Fundam. Appl. Toxicol.

[28] Hymery N, Sibiril Y, Parent-Massin D (2006). Improvement of human dendritic cell culture for immunotoxicological investigations. Cell Biol. Toxicol.

[29] Wetmore L, Nance DM (1991). Differential and sex-specific effects of kainic acid and domoic acid lesions in the lateral septal area of rats on immune function and body weight regulation. Exp. Neurol.

[30] Peng YG, Ramsdell JS (1996). Brain Fos induction is a sensitive biomarker for the lowest observed neuroexcitatory effects of domoic acid. Fundam. Appl. Toxicol.

[31] Maucher JM, Ramsdell JS (2005). Ultrasensitive detection of domoic acid in mouse blood by competitive ELISA using blood collection cards. Toxicon.

[32] Ehrchen J, Steinmuller L, Barczyk K, Tenbrock K, Nacken W, Eisenacher M, Nordhues U, Sorg C, Sunderkotter C, Roth J (2007). Glucocorticoids induce differentiation of a specifically activated, anti-inflammatory subtype of human monocytes. Blood.

[33] Weller RO, Engelhardt B, Phillips MJ (1996). Lymphocyte targeting of the central nervous system: a review of afferent and efferent CNS-immune pathways. Brain Pathol.

[34] Gill SS, Hou Y, Ghane T, Pulido OM (2008). Regional susceptibility to domoic acid in primary astrocyte cells cultured from the brain stem and hippocampus. Mar. Drugs.

[35] Calabrese E (2008). Hormesis: Why it is Important to Toxicology and Toxicologists. Environ. Toxicol. Chem..

[36] Calabrese E (2005). Hormetic Dose-Response Relationships in Immunology: Occurrence, Quantitative Features of the Dose Response, Mechanistic Foundations, and Clinical Implications. Crit. Rev. Toxicol.

[37] Mori C, Morsey B, Levin M, Nambiar PR, De Guise S (2006). Immunomodulatory effects of in vitro exposure to organochlorines on T-cell proliferation in marine mammals and mice. J. Toxicol. Environ. Health A.

[38] Kannurpatti SS, Joshi PG, Joshi NB (2000). Calcium sequestering ability of mitochondria modulates influx of calcium through glutamate receptor channel. Neurochem. Res.

[39] Peng TI, Greenamyre JT (1998). Privileged access to mitochondria of calcium influx through N-methyl-D-aspartate receptors. Mol. Pharmacol.

[40] Peng TI, Jou MJ, Sheu SS, Greenamyre JT (1998). Visualization of NMDA receptor-induced mitochondrial calcium accumulation in striatal neurons. Exp. Neurol.

[41] Hishikawa T, Cheung JY, Yelamarty RV, Knutson DW (1991). Calcium transients during Fc receptor-mediated and nonspecific phagocytosis by murine peritoneal macrophages. J. Cell Biol.

[42] Lew DP, Andersson T, Hed J, Di Virgilio F, Pozzan T, Stendahl O (1985). Ca2+-dependent and Ca2+-independent phagocytosis in human neutrophils. Nature.

[43] Christensen RN, Ha BK, Sun F, Bresnahan JC, Beattie MS (2006). Kainate induces rapid redistribution of the actin cytoskeleton in ameboid microglia. J. Neurosci. Res..

[44] Reynaud S, Duchiron C, Deschaux P (2003). 3-methylcholanthrene inhibits lymphocyte proliferation and increases intracellular calcium levels in common carp (Cyprinus carpio L). Aquat. Toxicol.

[45] Poulopoulou C, Markakis I, Davaki P, Nikolaou C, Poulopoulos A, Raptis E, Vassilopoulos D (2005). Modulation of voltage-gated potassium channels in human T lymphocytes by extracellular glutamate. Mol. Pharmacol.

[46] Webster JI, Tonelli L, Sternberg EM (2002). Neuroendocrine regulation of immunity. Annu. Rev. Immunol.

[47] Qiu S, Curras-Collazo MC (2006). Histopathological and molecular changes produced by hippocampal microinjection of domoic acid. Neurotoxicol. Teratol.

[48] Jakobsen B, Tasker A, Zimmer J (2002). Domoic acid neurotoxicity in hippocampal slice cultures. Amino Acids.

[49] Umegaki H, Yamamoto A, Suzuki Y, Iguchi A (2006). Stimulation of the hippocampal glutamate receptor systems induces stress-like responses. Neuro. Endocrinol. Lett.

[50] Petroni KC, Shen L, Guyre PM (1988). Modulation of human polymorphonuclear leukocyte IgG Fc receptors and Fc receptor-mediated functions by IFN-gamma and glucocorticoids. J. Immunol.

[51] Long F, Wang YX, Liu L, Zhou J, Cui RY, Jiang CL (2005). Rapid nongenomic inhibitory effects of glucocorticoids on phagocytosis and superoxide anion production by macrophages. Steroids.

[52] Goulding NJ, Euzger HS, Butt SK, Perretti M (1998). Novel pathways for glucocorticoid effects on neutrophils in chronic inflammation. Inflamm. Res.

[53] Goulding NJ, Euzger HS, Butt SK, Perretti M (1998). Novel pathways for glucocorticoid effects on neutrophils in chronic inflammation. Inflamm. Res..

[54] De A, Blotta HM, Mamoni RL, Louzada P, Bertolo MB, Foss NT, Moreira AC, Castro M (2002). Effects of dexamethasone on lymphocyte proliferation and cytokine production in rheumatoid arthritis. J. Rheumatol..

[55] Musiani M, Gentile L, Valentini M, Modesti A, Musiani P (1998). Lymphocyte proliferative response in brown bears: cytokine role and glucocorticoid effect. J. Exp. Zool.

[56] Ryan JC, Morey JS, Ramsdell JS, Van Dolah FM (2005). Acute phase gene expression in mice exposed to the marine neurotoxin domoic acid. Neuroscience.

[57] Germolec DR (2004). Sensitivity and predictivity in immunotoxicity testing: immune endpoints and disease resistance. Toxicol. Lett.

[58] Hermanowicz A, Nawarska Z, Borys D, Maslankiewicz A (1982). The neutrophil function and infectious diseases in workers occupationally exposed to organochloride insecticides. Int. Arch. Occup. Environ. Health.

[59] Loose LD, Silkworth JB, Charbonneau T, Blumenstock F (1981). Environmental chemical-induced macrophage dysfunction. Environ. Health Perspect.

[60] Cotran RS, Kumar V, Collins T (1999). Robbins pathologic basis of disease.

[61] Kuby J (1997). Immunology.

[62] Rzeski W, Ikonomidou C, Turski L (2002). Glutamate antagonists limit tumor growth. Biochem. Pharmacol.

[63] Stepulak A, Sifringer M, Rzeski W, Brocke K, Gratopp A, Pohl EE, Turski L, Ikonomidou C (2007). AMPA antagonists inhibit the extracellular signal regulated kinase pathway and suppress lung cancer growth. Cancer Biol. Ther..

[64] Carvalho PS, Catian R, Moukha S, Matias WG, Creppy EE (2006). Comparative study of Domoic Acid and Okadaic Acid induced-chromosomal abnormalities in the Caco-2 cell line. Int. J. Environ. Res. Public Health.

[65] Mathew A, Kurane I, Green S, Vaughn DW, Kalayanarooj S, Suntayakorn S, Ennis FA, Rothman AL (1999). Impaired T cell proliferation in acute dengue infection. J. Immunol.

[66] Brousseau P, Payette Y, Tryphonas H, Blakley B, Boermans H, Flipo D, Fournier M (1999). Manual of Immunological Methods.

[67] Brousseau P, Pellerin J, Morin Y, Cyr D, Blakley B, Boermans H, Fournier M (2000). Flow cytometry as a tool to monitor the disturbance of phagocytosis in the clam Mya arenaria hemocytes following in vitro exposure to heavy metals. Toxicology.

[68] Burleson GR, Dean JH, Munson AE (1995). Methods in Immunotoxicoogy.

[69] Suzuki CA, Hierlihy SL (1993). Renal clearance of domoic acid in the rat. Food Chem. Toxicol..

